# Hyperuricemia Is Independently Associated with Coronary Heart Disease and Renal Dysfunction in Patients with Type 2 Diabetes Mellitus

**DOI:** 10.1371/journal.pone.0027817

**Published:** 2011-11-18

**Authors:** Hiroyuki Ito, Mariko Abe, Mizuo Mifune, Koshiro Oshikiri, Shinichi Antoku, Yuichiro Takeuchi, Michiko Togane

**Affiliations:** Department of Diabetes, Metabolism and Kidney Disease, Edogawa Hospital, Tokyo, Japan; Universita Magna-Graecia di Catanzaro, Italy

## Abstract

**Aims:**

To investigate the relationship between hyperuricemia (HUA) and the clinical backgrounds in Japanese patients with type 2 diabetes mellitus.

**Methods:**

After a cross-sectional study evaluating the association of HUA with the clinical characteristics in 1,213 patients with type 2 diabetes mellitus, the estimated glomerular filtration rate (eGFR) and the incidence of diabetic macroangiopathies was investigated in a prospective observational study in 1,073 patients during a 3.5 year period. HUA was defined by serum uric acid levels >327 μmol/L or as patients using allopurinol.

**Results:**

The frequency of HUA was significantly higher in the diabetic patients (32% in men and 15% in women) than in the normal controls (14% in men and 1% in women). In total, HUA was found in 299 (25%) of the patients during the cross-sectional study. Even after adjusting for sex, drinking status, treatment for diabetes mellitus, body mass index, hypertension, use of diuretics, hyperlipidemia, HbA1c and/or the eGFR, the HUA was independently associated with some diabetic complications. The eGFR was significantly reduced in HUA patients compared to those with normouricemia in the 12 months after observation was started. HUA was also an independent risk factor for coronary heart disease even after adjustment in the Cox proportional hazard model.

**Conclusions:**

HUA is a associated with diabetic micro- and macroangiopathies. HUA is a predictor of coronary heart disease and renal dysfunction in patients with type 2 diabetes mellitus. However, the influence of HUA is considered to be limited.

## Introduction

It has been reported that hyperuricemia (HUA) is an independent risk factor for the progression of renal dysfunction [1], [2] and cardiovascular events [1]–[10], as well as gout. Type 2 diabetes mellitus is also well-known as a major risk factor for chronic kidney disease (CKD) and atherosclerotic disease. Although the serum uric acid level was previously shown to be higher in patients with diabetes mellitus than in the population without glucose intolerance [10], it was also elevated in the individuals with impaired glucose tolerance other than type 2 diabetes [11]–[14]. However, HUA has been reported to be associated with the onset of diabetes mellitus or metabolic syndrome [15]–[20]. Although several studies have reported the relationship between HUA and diabetic macroangiopathies, such as coronary heart disease, stroke and peripheral arterial disease, the conclusions have been controversial [21]–[27]. Furthermore, the changes in kidney function associated with the HUA have not yet been sufficiently studied in the patients with type 2 diabetes mellitus.

The aim of this study was to investigate the relationship between the HUA and the clinical background including diabetic complications and the glomerular filtration rate (GFR), in a cross-sectional study, and to determine the incidences of diabetic macroangiopathies and the changes in the eGFR according to a prospective observational study in Japanese patients with type 2 diabetes mellitus.

## Methods

### Ethics statement

This study was conducted according to the principles expressed in the Declaration of Helsinki. The Ethics Committees of Edogawa Hospital approved the protocol of this study and waived the need for written informed consent because the data were analyzed anonymously for this observation study based on the data stored in the hospital database.

### Study population and methods

First, a cross-sectional study was performed in a population of 1,213 patients diagnosed with type 2 diabetes mellitus who underwent consecutive evaluations, including urinalysis, and determination of the serum creatinine and uric acid levels in the Department of Diabetes, Metabolism and Kidney Disease of Edogawa Hospital, Tokyo, Japan between April 2008 and March 2009. The patients with end-stage renal disease receiving maintenance dialysis were excluded from this study. Subjects who underwent physical health examinations were also entered into the study as normal controls (age-matched, 156 men and 116 women). The control did not have diabetes mellitus or a history of myocardial and/or cerebral infarction.

Second, a prospective observational study was performed in the population of the cross-sectional study. The estimated GFR (eGFR) and new onset of diabetic micro- and macroangiopathies were estimated in 1,073 of the patients with type 2 diabetes mellitus who were treated for more than one year. All of the indications for anti-diabetic, anti-hypertensive and anti-hyperlipidemic agents were determined by each patient's physician during the observation period. The patients visited our hospital once every 1 to 4 months and the mean follow-up period was 28±8 months.

The eGFR was calculated using the formula reported by Matsuo *et al* [28]. This equation originated from the MDRD study group [29] arranged for Japanese individuals, and it is recommended by the Japanese Society of Nephrology: eGFR (mL/min/1.73 m^2^)  = 194×Scr^−1.094^×Age^−0.287^×0.739 (if female). The urinary albumin excretion (UAE) is presented as the albumin-to-creatinine ratio (ACR; mg/g creatinine). The ACR was staged according to an analysis of a spot urine sample as: stage I (normoalbuminuria), ACR<30 mg/g creatinine; stage II (microalbuminuria), 30≤ACR<300 mg/g creatinine; stage III (macroalbuminuria), ACR≥300 mg/g creatinine (or dipstick urinalysis revealed 2+, 3+ or 4+) and eGFR≥ 30 mL/min/1.73 m^2^; and stage IV, ACR≥ 300 mg/gcreatinine (or dipstick urinalysis revealed 2+, 3+ or 4+) and eGFR<30 mL/min/1.73 m^2^. Diabetic nephropathy included stages II, III and IV. Any individuals who had additional kidney diseases, such as acute renal failure and chronic glomerulonephritis, were excluded from this study.

The obese and non-obese individuals were defined as those having a BMI ≥ 25 kg/m^2^ and a BMI <25 kg/m^2^, respectively. The blood pressure was measured twice with the subjects in the sitting position after a 5 minute rest. The lower value of the two measurements was used for the study. Hypertension was defined as a systolic blood pressure≥140 mmHg and/or a diastolic blood pressure≥90mmHg. The participants currently using antihypertensive medications were also classified as positive for hypertension.

The serum total cholesterol, LDL-cholesterol, HDL-cholesterol and uric acid concentrations were measured with a TBA-200 FR NEO using Determiner L TC II, Determiner L LDL-C, Determiner L HDL-C and Determiner L UA instruments (Kyowa Medex Co., Ltd., Tokyo, Japan). Hyperlipidemia was defined by serum concentrations of total cholesterol ≥5.7 mmol/L, LDL-cholesterol levels ≥3.6 mmol/L, or as patients who were already undergoing treatment with lipid-lowering agents. The triglyceride concentrations were not investigated in this study because fasting blood samples could not always be obtained for measurements. HUA was defined by serum uric acid levels >327 μmol/L or as patients using allopurinol according to the guidelines proposed by the Japanese Society of Gout and Nucleic Acid Metabolism [30]. The HbA1c levels were determined by a high performance liquid chromatography method using an automated HLC-723G7 analyzer (Tosoh Corporation, Tokyo, Japan) and calibrated by the Japan Diabetes Society (JDS) standard calibrators. The value for HbA1c (%) is estimated as a National Glycohemoglobin Standardization Program (NGSP) equivalent value (%) calculated by the formula: HbA1c  =  HbA1c (JDS) + 0.4, considering the relational expression of HbA1c (JDS) measured by the previous Japanese standard substance and measurement methods and HbA1c (NGSP) [31].

Diabetic retinopathy was defined as simple, pre-proliferative and proliferative retinopathy judged according to the results of a funduscopic examination performed by expert ophthalmologists. Diabetic neuropathy was diagnosed by the presence of two or more components among clinical symptoms (bilateral spontaneous pain, hypoesthesia, or paraesthesia of the legs), the absence of ankle tendon reflexes and decreased vibration sensations using a C128 tuning fork according to the guidelines published by the JDS [32]. Cerebrovascular disease was diagnosed by the physicians as a history of an ischemic stroke using brain computed tomography or magnetic resonance imaging. Only the patients with symptoms were classified as having cerebrovascular disease, and cases of silent brain infarction, transient ischemic attack and brain hemorrhage were excluded from this study. Coronary heart disease was diagnosed based on a previous history of myocardial infarction, angina pectoris, electrocardiogram abnormalities suggesting myocardial ischemia or interventions after coronary angiographic examination. Peripheral arterial disease was diagnosed by the absence of a pulse in the legs along with ischemic symptoms, obstructive findings on ultrasonographic or angiographic examinations of the lower extremities, or an ankle-brachial pressure index (ABI) <0.9.

The ABI and brachial-ankle pulse wave velocity (baPWV) as indicators of atherosclerosis were measured using a Form PWV/ABI, BP-203PRE II instrument (Omron Colin Co., Ltd, Bunkyo, Tokyo, Japan). The intima-media thickness (IMT) of the carotid artery was measured via ultrasonographic examinations by skilled laboratory technicians using an Aplio XV ultrasound machine (Toshiba Medical Systems Corp., Ohtawara, Tochigi, Japan) as described previously [33].

### Statistical methods

All data are shown as the means ± SD. An analysis of variance (ANOVA) and the χ2 test were used for between-group comparisons of the continuous and categorical variables, respectively. A multiple logistic regression analysis was performed to determine the association of HUA with the other clinical parameters. Odds ratios (*OR*) and respective 95% confidence intervals (95% *CI*) were determined to examine the strength of the relationship between the HUA and the prevalence of diabetic micro- and macroangiopathies. The cumulative incidence of diabetic macroangiopathies was estimated by the Kaplan-Meier method, and the differences were assessed with the log-rank test. We used the Cox proportional hazard model to estimate the hazard ratio (*HR*) of HUA along with the 95% *CI*. Differences of *P*<0.05 (two-tailed) were considered to be statistically significant. The statistical software package JMP, version 8.0 (SAS Institute, Cary, NC, USA), was used to perform all of the analyses.

## Results

The mean uric acid concentration was not significantly different between the study subjects (321±83 μmol/L in men and 286±71 μmol/L in women) and normal controls (333±71 μmol/L in men and 274±48 μmol/L in women). However, the frequency of HUA was significantly higher in the patients (32% in men and 15% in women) than in the normal controls (14% in men and 1% in women, *P*<0.01). In total, HUA was found in 299 (25%) of the patients with type 2 diabetes mellitus. Allopurinol was administered to 273 of the patients with HUA. None of the patients received probenecid, benzbromarone and/or febuxostat for the treatment of HUA.

**Table 1** shows the clinical characteristics and the laboratory parameters of the patients. **Table 2** shows a comparison of clinical parameters between the groups with and without HUA. HUA was significantly more common in men, drinkers, obese subjects and those with hypertension than in those without. Patients receiving insulin or diuretic treatments also were more likely to have HUA, while those with hyperlipidemia were less likely to have HUA. The levels of HbA1c, serum HDL-cholesterol, creatinine, eGFR, and ABI were significantly lower in the subjects with HUA than in those without. The baPWV was elevated in the patients with HUA. Diabetic retinopathy and nephropathy were more common in the patients with HUA than in those without. Diabetic macroangiopathies were also frequently found in the subjects with HUA.

**Table 1:** The clinical characteristics of the patients.

	%/Mean ± SD	Number estimated (%)
Age (years)	64±12	1213 (100)
Men	59	1213 (100)
Duration of diabetes mellitus (years)	10±10	935 (77)
Current plus past smoking	59	780 (64)
Drinkers #	43	888 (73)
Treatment for diabetes mellitus		
Diet only/OHA/insulin	11/60/29	1213 (100)
Body mass index (kg/m^2^)	24.7±4.1	1192 (98)
Obesity ##	42	1192 (98)
Hypertension	73	1213 (100)
Anti-hypertensive agents		1213 (100)
ACEi	12	
ARB	43	
CCB	41	
Diuretics	10	
Hyperlipidemia	65	1212 (10)
HbA1c (%)	7.8±1.8	1122 (93)
Total cholesterol (mmol/L)	5.1±1.1	1119 (92)
LDL-cholesterol (mmol/L)	3.0±0.9	647 (53)
HDL-cholesterol (mmol/L)	1.5±0.5	816 (67)
Serum creatinine (μmol/L)	85±47	1213 (100)
Estimated GFR (mL/min/1.73 m^2^)	53±19	1213 (100)
CKD stage		1213 (100)
Stage 1+2	32	
Stage 3	59	
Stage 4+5	9	
Serum uric acid (μmol/L)	307±81	1213 (100)
Diabetic retinopathy $	41	889 (73)
Diabetic neuropathy	75	864 (71)
Diabetic nephropathy		111 (92)
Stage I	60	
Stage II	18	
Stage III+IV	21	
Cerebrovascular disease	14	1208 (100)
Coronary heart disease	22	1209 (100)
Peripheral arterial disease	5	1211 (100)
ABI	1.1±0.1	636 (52)
baPWV (cm/s)	1769±398	633 (52)
Carotid IMT (mm)	1.0±0.3	359 (30)

OHA: oral hypoglycemic agents, ACEi: angiotensin-converting enzyme inhibitor, ARB: angiotensin II receptor blocker, CCB: calcium channel blocker, GFR: glomerular filtration rate, CKD: chronic kidney disease, ABI: ankle-brachial index, baPWV: brachial-ankle pulse wave velocity, and IMT: intima-media thickness

# Drinkers were defined as those who consumed more than 20 g/day of ethanol.

## Obesity was considered to be present in individuals with a body mass index ≥25 kg/m^2^.

$ Diabetic retinopathy includes simple, pre-proliferative, and proliferative retinopathies.

**Table 2:** A comparison of the clinical parameters between groups without and with hyperuricemia.

	%/Mean ± SD		
	Normouricemia	Hyperuricemia	*P*
	(*n* = 914)	(*n* = 299)	
Age (years)	64±12	65±12	0.34
Men	54	76	<0.01
Duration of diabetes mellitus (years)	10±10	11±10	0.09
Current plus past smoking	58	61	0.55
Drinkers #	41	50	0.02
Treatment for diabetes mellitus			
Diet only/OHA/insulin	11/63/26	10/52/38	<0.01
Body mass index (kg/m^2^)	24.4±4.0	25.7±4.4	<0.01
Obesity ##	39	52	<0.01
Hypertension	69	87	<0.01
Use of diuretics	4	27	<0.01
Hyperlipidemia	67	59	0.01
HbA1c (%)	7.9±1.9	7.6±1.7	0.03
Total cholesterol (mmol/L)	5.1±1.0	5.0±1.2	0.10
LDL-cholesterol (mmol/L)	3.0±0.9	3.0±1.0	0.41
HDL-cholesterol (mmol/L)	1.6±0.1	1.4±0.4	<0.01
Serum creatinine (μmol/L)	71±27	115±80	<0.01
Estimated GFR (mL/min/1.73 m^2^)	60±17	40±17	<0.01
CKD stage			<0.01
Stage 1+2	40	9	
Stage 3	57	64	
Stage 4+5	3	27	
Serum uric acid (μmol/L)	283±64	378±85	<0.01
Diabetic retinopathy $	37	54	<0.01
Diabetic nephropathy $$	33	64	<0.01
Diabetic neuropathy	74	75	0.19
Cerebrovascular disease	12	19	<0.01
Coronary heart disease	20	27	<0.01
Peripheral arterial disease	4	7	0.01
ABI	1.11±0.11	1.08±0.15	<0.01
baPWV (cm/s)	1747±357	1835±506	0.02
Carotid IMT (mm)	1.0±0.2	1.0±0.3	0.89

OHA: oral hypoglycemic agents, ACEi: angiotensin-converting enzyme inhibitor, ARB: angiotensin II receptor blocker, CCB: calcium channel blocker, GFR: glomerular filtration rate, CKD: chronic kidney disease, ABI: ankle-brachial index, baPWV: brachial-ankle pulse wave velocity, and IMT: intima-media thickness

# Drinkers were defined as those who consumed more than 20 g/day of ethanol.

## Obesity was considered to be present in individuals with a body mass index ≥25 kg/m^2^.

$ Diabetic retinopathy includes simple, pre-proliferative, and proliferative retinopathies.

$$ Diabetic nephropathy includes ACR stages II, III, and IV.

The odd ratios for diabetic micro- and macroangiopathies with HUA as determined by a logistic regression analysis are shown in **Table 3**. HUA was significantly associated with diabetic angiopathies, excluding neuropathy. However, HUA was associated with only diabetic retinopathy and nephropathy after adjustment for sex, drinking status, treatment for diabetes mellitus, body mass index, hypertension, use of diuretics, hyperlipidemia, HbA1c and/or the eGFR.

**Table 3:** The odds ratios for the diabetic micro- and macroangiopathies in patients with hyperuricemia determined by the logistic regression analyses.

	Wald χ2 score	*OR* (95% CI)	*P*
Unadjusted			
Retinopathy $	21.6	2.06 (1.52–2.79)	<0.01
Nephropathy $$	77.9	3.75 (2.80–5.04)	<0.01
Neuropathy	1.7	1.28 (0.89–1.86)	0.19
CVD	9.4	1.73 (1.21–2.45)	<0.01
CHD	6.6	1.49 (1.10–2.01)	0.01
PAD	4.2	1.80 (1.01–3.14)	0.04
Adjusted (model 1)			
Retinopathy $	3.3	1.50 (0.97–2.33)	0.07
Nephropathy $$	13.2	2.12 (1.42–3.19)	<0.01
Neuropathy	2.3	0.69 (0.40–1.13)	0.13
CVD	0.2	0.89 (0.51–1.50)	0.66
CHD	2.0	0.71 (0.45–1.13)	0.15
PAD	0.0	1.00 (0.38–2.48)	0.99
Adjusted (model 2)			
Retinopathy $	5.5	1.65 (1.09–2.51)	0.02
Nephropathy $$	27.1	2.79 (1.90–4.12)	<0.01
Neuropathy	1.1	0.77 (0.48–1.27)	0.30
CVD	0.0	0.97 (0.58–1.59)	0.90
CHD	0.2	0.90 (0.58–1.37)	0.61
PAD	0.3	1.27 (0.52–2.93)	0.58
Adjusted (model 3)			
Retinopathy $	3.8	1.66 (0.99–2.78)	0.05
Nephropathy $$	23.4	3.40 (2.08–5.62)	<0.01
Neuropathy	0.5	0.80 (0.44–1.47)	0.46
CVD	0.1	0.90 (0.43–1.79)	0.76
CHD	0.5	0.81 (0.46–1.41)	0.47
PAD	1.1	1.72 (0.83–2.90)	0.29

CVD: cerebrovascular disease, CHD: coronary heart disease, PAD: peripheral arterial disease

$ Diabetic retinopathy includes simple, pre-proliferative, and proliferative retinopathies.

$$ Diabetic nephropathy includes ACR stages II, III, and IV.

Model 1 was adjusted for sex, drinking status, treatment for diabetes mellitus, body mass index, hypertension, use of diuretics, hyperlipidemia, HbA1c and the eGFR.

Model 2 was adjusted for sex, drinking status, treatment for diabetes mellitus, body mass index, use of diuretics, hyperlipidemia and HbA1c.

Model 3 was adjusted for age, sex, duration of diabetes, smoking status, drinking status, treatment for diabetes mellitus, body mass index, use of diuretics, hyperlipidemia and HbA1c.

**Table 4** shows the baseline characteristics of the patients with type 2 diabetes mellitus for the prospective observation study. The mean serum uric acid concentration was 303±77 μmol/L, and HUA was found in 202 (32%) of men and 60 (14%) of women. Allopurinol was administered to 246 patients with HUA.

**Table 4:** The baseline characteristics of the patients evaluated in the observation study.

	%/Mean ± SD	Number estimated (%)
Age (years)	64±12	1073 (100)
Men	59	1073 (100)
Drinkers #	43	888 (73)
Body mass index (kg/m^2^)	24.7±4.0	1054 (98)
Obesity ##	42	1054 (98)
Hypertension	73	1073 (100)
Hyperlipidemia	66	1072 (10)
HbA1c (%)	7.7±1.7	994 (93)
Serum creatinine (μmol/L)	80±44	1073 (100)
Estimated GFR (mL/min/1.73 m^2^)	54±18	1073 (100)
Serum uric acid (μmol/L)	303±77	1073 (100)
Cerebrovascular disease	14	1070 (100)
Coronary heart disease	22	1070 (100)
Peripheral arterial disease	5	1073 (100)

GFR: glomerular filtration rate.

# Drinkers were defined as those who consumed more than 20 g/day of ethanol.

## Obesity was considered to be present in individuals with a body mass index ≥25 kg/m^2^.

**Figure 1:**
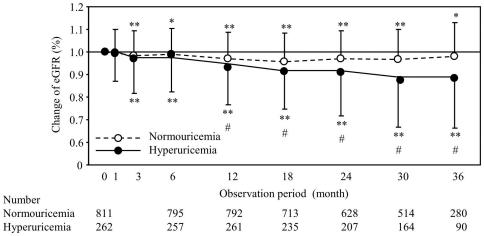
The changes in the eGFR in the patients with and without hyperuricemia. The data represent the means ± SD. Open and closed circles represent the values in normouricemic and hyperuricemic individuals, respectively. * *P*<0.05 and ** *P*<0.01 vs. 0 month. # <0.01 vs. normouricemia.

**Figure 1** shows the changes of the eGFR in patients without and with HUA. The eGFR was significantly reduced in the patients with HUA compared to those without HUA in during the 12 months after the observation was started.

**Figure 2:**
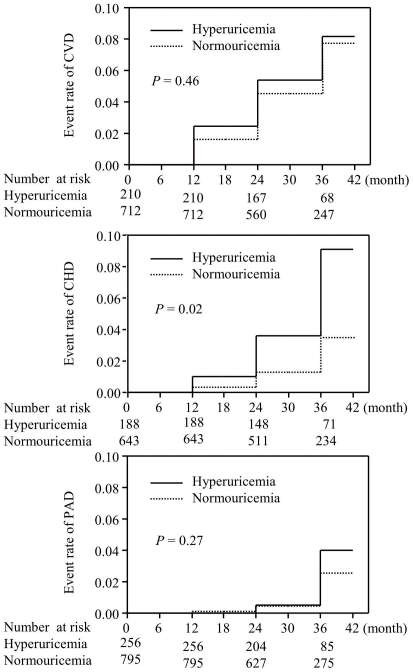
The incidences of diabetic macroangiopathies in the patients with and without hyperuricemia. CVD: cerebrovascular disease, CHD: coronary heart disease and PAD: peripheral arterial disease. Solid and dashed lines represent the incidences in hyperuricemic and normouricemic individuals, respectively. Although hyperuricemia did not affect the incidence of CVD or PAD, it was a significant risk factor for CHD.

The incidence of diabetic macroangiopathies in the groups without and with HUA is shown in **Figure 2**. Although HUA did not affect the incidence of CVD (*HR* 1.28, [95% *CI* 0.63–2.42], log-rank test, *P* = 0.46) and PAD (*HR* 1.76, [95% *CI* 0.59–4.79], log-rank test, *P* = 0.27), it was a significant risk factor for CHD (*HR* 2.60 [95% *CI* 1.11–5.92], log-rank test, *P* = 0.02).

In the Cox proportional hazard models, HUA was an independent risk factor (*HR* 2.81 [95% *CI* 1.00–7.81], *P* = 0.049) for coronary heart disease even after adjustment for sex, drinking status, treatment for diabetes mellitus, body mass index, hypertension, hyperlipidemia and the eGFR.

## Discussion

In the present study, the frequency of HUA was significantly higher in the patients with type 2 diabetes mellitus than in the normal subjects. The prevalence of HUA in patients with type 2 diabetes varied in the previous reports [10]–[14]. Fang *et al.* described that the serum uric acid level was higher in diabetic women than in non-diabetic women, although it was not different between diabetic and non-diabetic men, according to the National Health and Nutrition Examination Survey in the USA [10]. On the other hand, it was reported that HUA was more common in the patients with impaired glucose tolerance than in those with diabetes mellitus or normal subjects [11]–[13]. Alderman *et al*. reported that the serum uric acid concentration was not associated with the presence of diabetes mellitus [14], and Wen *et al*. described that it was negatively correlated with the blood glucose level in a population of 484,568 subjects in Taiwan [3]. Li *et al* also reported inverse correlations of the blood glucose and HbA1c levels with the serum uric acid concentration in patients with type 2 diabetes mellitus [34]. In the present study, the HbA1c level was also lower in patients with HUA than in those without, and it was negatively correlated with the serum uric acid concentration (HbA1c = −0.17 x uric acid level + 8.33, univariate analysis, *P*<0.01). Although these results seem to be conflicting, it is clear that HUA is a risk factor for diabetes mellitus and/or metabolic syndrome [15]–[20]. The discrepancies in the studies are considered to be caused by the effects of insulin on the renal proximal tubules. As a likely mechanism linking HUA and diabetes mellitus/metabolic syndrome, it is known that hyperinsulinemia reduces the urinary excretion of uric acid by activating the transporter of uric acid (URAT), which is expressed in the proximal tubules of the kidneys [35], [36]. Exogenous insulin also suppresses urinary uric acid excretion [37], [38]. Although the serum insulin or C-peptide concentration was not evaluated in the present study, frequent insulin treatment (29%) in addition to the presence of obesity (42%), which is often present in patients with endogenous hyperinsulinemia, may have increased the prevalence of HUA in our study group. Therefore, body mass index and insulin use of the study subjects should be considered when the prevalence of HUA is discussed in patients with type 2 diabetes mellitus.

The relationship between vascular diseases and HUA in patients with type 2 diabetes has not been fully explored. In our cross-sectional study, HUA was associated with diabetic micro- and macroangiopathies, as well as the ABI and baPWV, which are surrogate markers for atherosclerosis (**Table 2**). Furthermore, the incidence of coronary heart disease was significantly higher in the patients with HUA than in those without during the follow-up observation period (**Figure 2**). Rathmann *et al*. reported that HUA was associated with coronary heart disease in 4,047 patients with type 2 diabetes mellitus according to a cross-sectional study [21]. Several other investigators have described elevated serum uric acid to be a risk factor for atherosclerotic disease in patients with diabetes mellitus [22]–[26]. In contrast, Ong *et al*. recently reported that the serum uric acid level did not predict cardiovascular mortality in 1,268 patients with type 2 diabetes in western Australia [27]. All of these studies determined the incidence of vascular events after dividing the subjects into tertiles, quartiles or quintiles according to their initial level of serum uric acid. In the present study, subjects were divided into two groups that were independent other than their uric acid levels. When allopurinol was used, an individual was defined as having HUA even if the serum uric acid level was within the normal range. Therefore, our study investigated the association between diabetic complications and the existence of HUA, but not the serum uric acid levels. Our data suggested that HUA clearly predicted the incidence of diabetic macroangiopathies in the patients with type 2 diabetes mellitus, but that its influence was limited. However, the relatively short duration of follow-up in our study might have led us to underestimate the incidence of vascular events, even though our study included a large number of patients.

It has been established that hyperuricemia is an independent risk factor for the progression of renal dysfunction [1], [2]. However, the influence of HUA on the renal functions has been insufficiently investigated in patients with diabetes mellitus. In the present study, the eGFR significantly decreased in the patients with HUA compared with those without. Tseng *et al*. [39] and Fukui *et al*. [40] reported that the serum uric acid level was elevated, along with increased urinary albumin excretion in the smaller study population with type 2 diabetes using a cross-sectional design. Li *et al*. [31] revealed that the level of serum uric acid was negatively correlated with the eGFR in 1,026 Chinese patients with type 2 diabetes mellitus. However, our study is the first report investigate the changes in the eGFR between diabetic patients without and with HUA. It is considered to be important that a significant difference in the glomerular function between the two groups was observed within one year after observation was started.

The ABI, baPWV and carotid IMT are known to be surrogate markers for atherosclerosis. They show abnormal values in patients with type 2 diabetes mellitus, as we previously reported [33], [40]. In the present study, the ABI was significantly lower and baPWV was higher in the patients with HUA than in those without. Funaki *et al*. also described significant correlations between the serum uric acid concentration and the ABI and carotid IMT in patients with type 2 diabetes [41]. Therefore, the HUA may reflect subclinical atherosclerosis, as well as previously diagnosed macroangiopathies, in patients with type 2 diabetes mellitus. Intensive examination to detect the vascular complications might be valuable in the clinic when HUA is found in patients with type 2 diabetes mellitus.

The present study has several important limitations. First, the follow-up period was relatively short compared to other studies. The number of patients decreased by nearly one-third at 36 months. This might cause us to underestimate the incidence of diabetic macroangiopathies. Second, the initial level of the eGFR was different between the groups without and with HUA. The levels should be similar in order to clarify the deterioration of the eGFR in the subjects with HUA and type 2 diabetes mellitus. Third, it was impossible to evaluate the effect of allopurinol administration for the normalization of the uric acid level, because allopurinol was used in most of the patients with HUA in our study. Recently, an anti-atherogenic effect of allopurinol was reported in patients with angina pectoris [42]. Therefore, future studies should closely examine the difference between patients treated with allopurinol and those who are untreated. Fourth, we did not evaluate the course of the serum uric acid levels and changes in the anti-diabetic, anti-hyperlipidemic and anti-hypertensive agents administered during the observation period. Changes in the levels of serum uric acid, blood glucose, lipids and blood pressure might therefore have affected the incidence of diabetic macroangiopathies in the present study. Fifth, the number of controls in this study was relatively small. Therefore, there may have been some selection bias. Sixth, the frequency of peripheral arterial disease seems to be low compared to the frequencies of cerebrovascular and coronary heart diseases. This might have been due to the small number of the patients who underwent ABI as a screening test. The underestimation of the prevalence and new onset of peripheral arterial disease should be considered for both the cross-sectional and the observational studies.

In conclusion, HUA is associated with diabetic micro- and macroangiopathies. Furthermore, HUA is a predictor of coronary heart disease and renal dysfunction in patients with type 2 diabetes mellitus. However, the influence of HUA was considered to be limited in our study during the relatively short observation period.
